# Predictors of psychosocial functioning in euthymic patients with bipolar disorder

**DOI:** 10.1192/j.eurpsy.2023.1489

**Published:** 2023-07-19

**Authors:** S. Ajmi, O. Sana, S. Najeh, F. Rim, G. Imen, C. Nada, B. T. Jihen, Z. Lobna, M. Mohamed, M. B. Manel

**Affiliations:** 1 Psychiatry C Department, Hedi chaker University hospital, sfax, Tunisia

## Abstract

**Introduction:**

Functional impairment is a major target in the treatment of bipolar disorder (BD), but the magnitude and type of functional difficulties differ across patients.

**Objectives:**

The aim of this study was to assess functioning and identify factors associated with global functioning in euthymic patients.

**Methods:**

It was a descriptive cross-sectional study. The population study consisted of patients diagnosed with BD (DSM 5), who were euthymic and followed up at the psychiatry department of CHU Hedi Chaker.

The Hamilton Depression Scale (HAM-D), the Young Mania Rating Scale (YMRS) and the Functioning Assessment Short Test (FAST) were used to assess depressive, manic symptoms and the functional impairment in bipolar patients respectively. All statistical analyses were performed using the SPSS software package v 18.

**Results:**

We collected 40 patients. They had an average age of 36 years and the sex ratio (M/F) was 1.

They had an educational level not exceeding primary studies in 46% of cases.

The average scores of HAM-D and YMRS were 4.57±4.58 and 3.43±2.89 respectively.

The average total functioning score of our patients was 19.13±16.5. Functional impairment was noted in 60% of them. The domains most affected were: occupational activity (62%), cognitive functioning (63%) and autonomy (50%). Fonctional impairement was associated with residual depressive and manic symptoms (p=0.013) and manic/hypomanic or depressive episodes with mixed features (p=0,005).

**Conclusions:**

Greater efforts should be directed toward targeting functioning in patient care, as it constitutes the most meaningful endpoint of response to treatment, especially with occupational and cognitive rehabilitation, thus allowing patients to overcome the course of illness and carry fulfilling lives.

**Disclosure of Interest:**

None Declared

